# Research on Palmprint Identification Method Based on Quantum Algorithms

**DOI:** 10.1155/2014/670328

**Published:** 2014-07-03

**Authors:** Hui Li, Zhanzhan Zhang

**Affiliations:** School of Electrical Engineering and Automation, Henan Polytechnic University, Jiaozuo 454010, China

## Abstract

Quantum image recognition is a technology by using quantum algorithm to process the image information. It can obtain better effect than classical algorithm. In this paper, four different quantum algorithms are used in the three stages of palmprint recognition. First, quantum adaptive median filtering algorithm is presented in palmprint filtering processing. Quantum filtering algorithm can get a better filtering result than classical algorithm through the comparison. Next, quantum Fourier transform (QFT) is used to extract pattern features by only one operation due to quantum parallelism. The proposed algorithm exhibits an exponential speed-up compared with discrete Fourier transform in the feature extraction. Finally, quantum set operations and Grover algorithm are used in palmprint matching. According to the experimental results, quantum algorithm only needs to apply square of *N* operations to find out the target palmprint, but the traditional method needs *N* times of calculation. At the same time, the matching accuracy of quantum algorithm is almost 100%.

## 1. Introduction 

**Figure 1:**
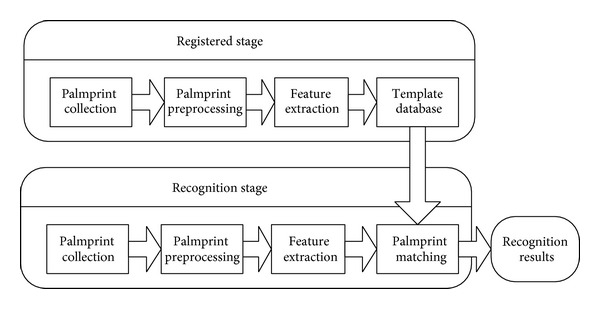
Flow chart of palmprint recognition.

Biological recognition technology is more and more important in this modern society [1]. Palmprint as a new biometric feature has several advantages compared to other available features: low-resolution images can be used, low cost capture devices can be used, it is very difficult or impossible to fake a palmprint, and the line features of the palmprints are stable, and so forth [2]. Zhang et al. summarized and compared the common palmprint recognition algorithms [3]. Jing et al. proposed an optimal subset-division based discrimination (OSDD) approach to enhance the classification performance of discriminant analysis technique [4]. It employs the kernel *K*-means algorithm to divide the sample set in the kernel space and obtains the nonlinear projection transformation. Guo et al. presented a study on feature band selection by analyzing hyperspectral palmprint data (520–1050 nm) [5]. Their research could be used as the guidance for designing new online multispectral palmprint systems. Palmprint recognition includes three important steps: palmprint preprocessing, feature extraction, and matching. Line feature extraction and line matching are proposed to detect whether a couple of palmprints are from the same palm. General recognition process has been shown in Figure 1.

Quantum computing and quantum information is a perfect product which combines quantum mechanics theory and classical computing theory. Quantum algorithm can solve some classical nonpolynomial problems in polynomial time and has many advantages of the superposition, coherence, and entanglement of the quantum state. So far, the most representative quantum algorithms are the large prime numbers factorization algorithm proposed by Shor [6] and the quantum searching algorithms in database proposed by Grover [7] in 1997. Grover algorithm is the kind of quantum searching method which can search a particular element in an unsorted database. This method can exponentially speed up the searching speed. Applying quantum algorithms to image processing is still in the stage of development; studies show that it is feasible to use quantum information and quantum computation in the image processing. Vlasov et al. [8] proposed a simple model which applied quantum computing method in the image recognition in 1997. In 2003, Schutzhold [9] pointed out that the specific mode could be identified and searched from the macrostructure images on a quantum computer. In May 2006, Pang et al. [10] proposed an algorithm which can successfully check out the best matching pattern between input image and sample collection. Subsequently, Pang [11, 12] proposed a series of quantum image compression algorithm. In this paper, quantum algorithms are used in the recognition steps including filtering processing, feature extraction, and palmprint matching. Every application step has been experimented and analyzed. According to the experiment results, applying quantum algorithm to palmprint recognition can decrease the numbers of repetition and raise the probability of success.

## 2. Palmprint Filtering Processing Based on Quantum Adaptive Median Filtering Algorithm

**Figure 2:**
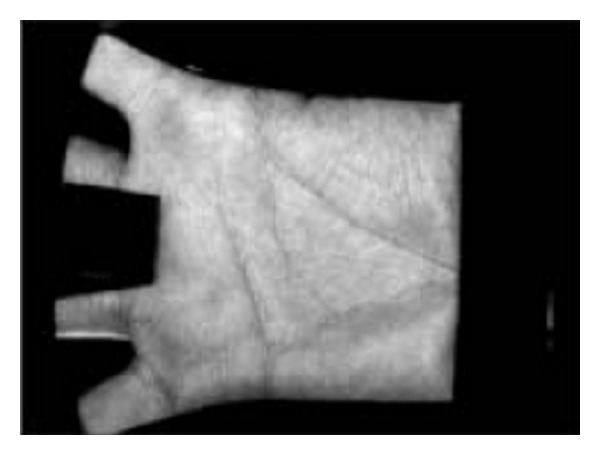
Original palmprint.

**Figure 3:**
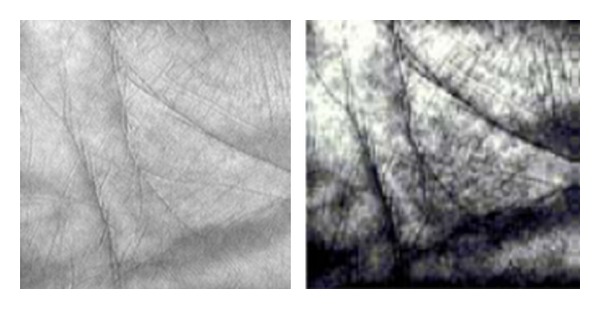
Segmented and normalized palmprint.

Before palmprint filter, an original palmprint is needed to be segmented and normalized. A palmprint is extracted from palmprint database which is shown in Figure 2. The palmprint after segmentation and normalization is shown in Figure 3.

Traditional gray pretreatment methods include histogram equalization, median filter, mean filter, and Gaussian filter [13]. In this paper we propose a quantum adaptive median filtering algorithm on palmprint pretreatment. Based on quantum measurements and collapse tenet, median filtering algorithm is applied on the framework of the quantum signal processing. It is an adaptive median filter because it can adaptively adjust the neighbor size and shape; it is based on the local features of translational position of operation template. The size is the number of elements and the shape is the distribution of elements.

For a normalized digital palmprint image *f*(*m*, *n*) ∈ [0,1], *f*(*m*, *n*) stands for the pixel gray value of this palmprint at the position of (*m*, *n*). At the same time *f*(*m*, *n*) and 1 − *f*(*m*, *n*), respectively, denote the probability when the pixel (*m*, *n*) gray-scale value is 1 and 0. Palmprint gray values of 1 and 0 can be represented by |0〉 and |1〉; the quantum bit form of the image *f*(*m*, *n*) is

(1) $$\begin{array}{c} |f\left(m,n\right)\rangle =\sqrt{1-f\left(m,n\right)}|0\rangle +\sqrt{f\left(m,n\right)}|1\rangle . \end{array}$$

Traditional filter window can be expressed as the following equation:

(2) $$\begin{array}{c} Wf\left(i,j\right)=[\begin{array}{ccccc} f_{i-n,j-n} & \ldots & f_{i-n,j} & \ldots & f_{i-n,j+n}\\ \ldots & \ldots & \ldots & \ldots & \ldots \\ \ldots & \ldots & f_{i,j} & \ldots & \ldots \\ \ldots & \ldots & \ldots & \ldots & \ldots \\ \ldots & \ldots & f_{i+n,j} & \ldots & f_{i+n,j+n} \end{array}], \end{array}$$

where *i* and *j* represent the *x*-axis and *y-*axis coordinates of the image. It can be translated into quantum bit form as

(3) $$\begin{array}{c} |Wf\left(i,j\right)\rangle =[\begin{array}{ccccc} |f_{i-n,j-n}\rangle & \ldots & |f_{i-n,j}\rangle & \ldots & |f_{i-n,j+n}\rangle \\ \ldots & \ldots & \ldots & \ldots & \ldots \\ \ldots & \ldots & |f_{i,j}\rangle & \ldots & \ldots \\ \ldots & \ldots & \ldots & \ldots & \ldots \\ \ldots & \ldots & |f_{i+n,j}\rangle & \ldots & |f_{i+n,j+n}\rangle \end{array}], \end{array}$$

where *f*(*m*, *n*) is transformed as *f*_*m*,*n*_ and |*f*(*m*, *n*)〉 ≥ |*f*_*m*,*n*_〉. We use a quantum Hadamard operation [14] in each pixel; the quantum Hadamard operation is

(4) $$\begin{array}{c} H=\frac{1}{\sqrt{2}}\left[\begin{array}{cc} 1 & 1\\ 1 & -1 \end{array}\right]. \end{array}$$

The operated results are

(5) |H·Wf(i,j)〉=[H·|fi−n,j−n〉…H·|fi−n,j〉…H·|fi−n,j+n〉…………………H·|fi,j〉………………………H·|fi+n,j〉…H·|fi+n,j+n〉],

where

(6) H·|fi,j〉=H·(ωi,j0|0〉+ωi,j1|1〉)=12(ωi,j0+ωi,j1)|0〉+12(ωi,j0−ωi,j1)|1〉.

The overall effect of formula (1) on formula (6) is

(7) $$\begin{array}{c} T_{wf}:Wf\left(i,j\right)⟶|HWf\left(i,j\right)\rangle . \end{array}$$

In other words, the gray values which are 1 and 0 in the traditional palmprint window are converted to

(8) $$\begin{array}{c} 0⟶\frac{1}{\sqrt{2}}|0\rangle +\frac{1}{\sqrt{2}}|1\rangle ,\\ 1⟶\frac{1}{\sqrt{2}}|0\rangle -\frac{1}{\sqrt{2}}|1\rangle . \end{array}$$

After all of the operations, the distribution of gray range in the palmprint image is compressed. It is conducive to generate the median operator template.

**Figure 4:**
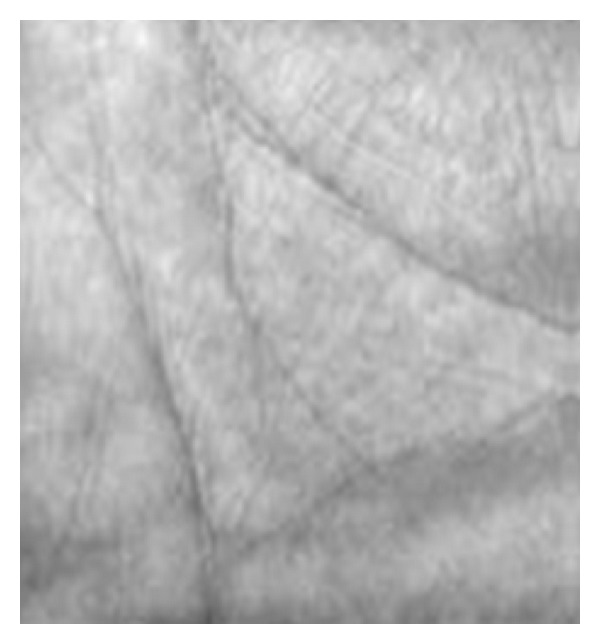
Effect of traditional adaptive median filtering algorithm.

**Figure 5:**
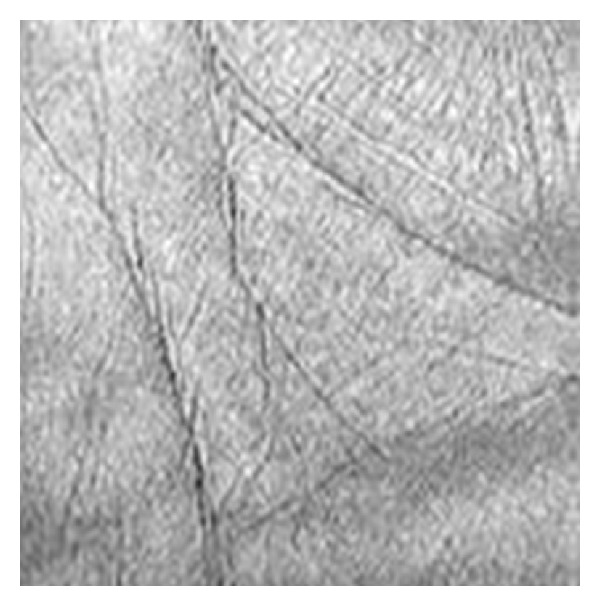
Effect of quantum adaptive median filtering algorithm.

Then *n* = *N* × *N*{*r*_*i*,*j*_ ∈ [0.5,1]} of numbers are randomly generated; if $r_{i,j}>\left(1/\sqrt{2}\right){\left(\omega_{i,j}^{0}+\omega_{i,j}^{1}\right)}^{2}$, then *H* · |*f*_*i*,*j*_〉 = 0 in formula (5); else *H* · |*f*_*i*,*j*_〉 = 1. Then we can get the readjusted binary window |*i*_*b*_(*i*, *j*)〉. Quantum adaptive median filter can be expressed as

(9) $$\begin{array}{c} y\left(i,j\right)⟹\text{Med}\left\{f_{i+r,j+s},\left(r,s\right)\in |i_{b}\left(i,j\right)\rangle \right\}, \end{array}$$

where  *r* and *s* belong to |*i*_*b*_(*i*, *j*)〉 and are the pixels located on *x*- and *y*-axes in binary window |*i*_*b*_(*i*, *j*)〉. Figures 4 and 5 are the comparison figures which are based on the filtering effect of adaptive median filtering algorithm and traditional adaptive median filtering algorithm.

Obviously, by using quantum adaptive median filtering algorithm, not only the image details can be better preserved but also the filtering ability is improved. Next we apply the binarization processing and pixel flip operation to filtered palmprint in order to benefit from the feature extraction.

## 3. Palmprint Feature Extraction Based on Quantum Fourier Transform

**Figure 6:**
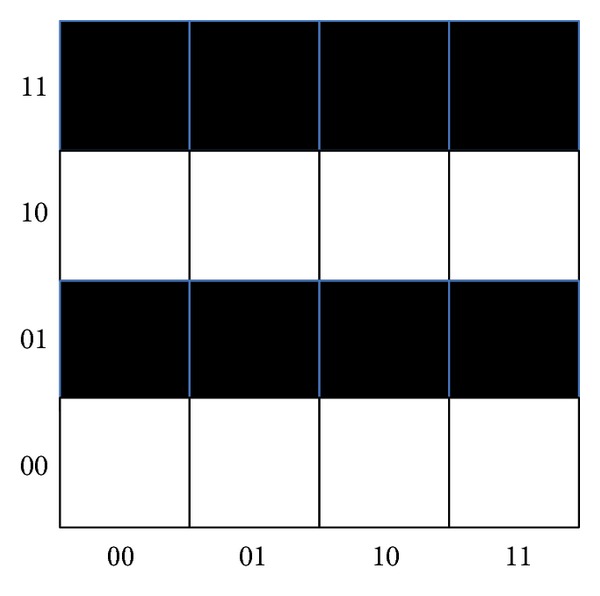
Fingerprint subgraph.

Figure 6 shows a palmprint image extracted after binarization figure (*N* × *N* dimensions) which has a lot of black spots and white dots after amplification; all white dots form a pattern or image. Our purpose is to extract the characteristic of fingerprint image, composed of all the white dots to match operation.

Traditional feature extraction method is mainly based on discrete Fourier transform. For a given *N* dimension the traditional discrete Fourier transform [15] is a linear operator on *C* ^*N*^ mapping (*x*_0_, *x*_1_,…, *x*_*N*−1_) to (*y*_0_, *y*_1_,…, *y*_*N*−1_), where

(10) $$\begin{array}{c} y_{k}=\frac{1}{\sqrt{N}}\overset{N-1}{\underset{i=0}{\sum }}x_{j}e^{2\pi ikj/N}\;\left(k=0,1,\ldots ,N-1\right).\; \end{array}$$

The quantum Fourier transform (QFT) algorithm can be obtained from the traditional discrete Fourier transform [16] which is

(11) $$\begin{array}{c} \text{QFT}:U_{\text{QFT}}|x\rangle =\frac{1}{\sqrt{2^{m}}}\overset{2^{m}-1}{\underset{i=0}{\sum }}e^{2\pi itx/2^{m}}|t\rangle . \end{array}$$

QFT can be used in novel image encryption and decryption [17], where the quantum bit numbers of the quantum state |*x*〉 are *m*, *U*_QFT_ is a unitary operator, and QFT is a 2^*m*^-dimensional unitary transformation [18, 19]. The effect of (11) is to transform a unit quantum state into a superposition state. After once QFT we can get multiple quantum states. Some coefficients quantum states are negative number and the others are positive number. The result of amplitude distribution shows significant concentration. When we measure the quantum state and then the state collapses, we can produce a better probability of success. Then we use QFT method to extract the palmprint feature.

The dimension of the filtered palmprint is *M* × *N* (*M* = 2^*a*^, *N* = 2^*b*^); we need to build *a* + *b* numbers of quantum registers; elements in quantum registers are *a* + *b* quantum bits on *C* ^*M*×*N*^ and are *M* × *N*-dimensional complex column vectors, and the location of the whiter point can be expressed by the coordinates of *x* and *y*. Let *z* = *x* + *ny*; *n* are the numbers of white points in each line, and |*z*〉 = |*x*〉 ⊗ |*y*〉. All the steps of our algorithm are as follows.

A quantum initial state is constructed which expresses the locations of all the white points in palmprint as

(12) $$\begin{array}{c} |\varphi \rangle =\frac{1}{\sqrt{\rho MN}}\overset{\rho MN}{\underset{k=1}{\sum }}|z_{k}\rangle , \end{array}$$

where *ρ* is the proportion of the white spot accounting for all pixels and is the white spots divided by the total number of black and white dots; then the QFT is applied to the state |*φ*〉:

(13) $$\begin{array}{c} U_{\text{QFT}}|\varphi \rangle =\overset{MN}{\underset{t=1}{\sum }}\overset{\rho MN}{\underset{k=1}{\sum }}\frac{1}{MN\sqrt{\rho }}e^{2\pi iz_{k}t/MN}|t\rangle . \end{array}$$

Formula (13) includes (*MN*) × (*ρ* *MN*) items after being expanded. After comparing with formula (10) and formula (13), we can find out the difference between traditional Fourier transform and QFT: traditional method is unitary transformation on *N*-dimensional Euclidean space, QFT is unitary transformation on *M* × *N*-dimensional space, and computation method of traditional method is serial computation, but QFT is parallel computing; complex vector (*x*_0_, *x*_1_,…, *x*_*N*−1_) has become one row of the quantum state matrix which facilitates high-speed processing. If we apply traditional feature extraction method on a palmprint image, the amount of computation will be very large. In this paper, we use the parallel computing features of the quantum algorithm; the (*MN*)×(*ρ* *MN*) numbers of calculations can be completed through using one time of Fourier transform operation. Compared with traditional algorithm, the calculation speed of our method shows exponential improvement. By using formula (13) in Figure 6,

(14) $$\begin{array}{c} U_{\text{QFT}}|\varphi \rangle =\overset{16}{\underset{t=1}{\sum }}\overset{8}{\underset{k=1}{\sum }}\frac{\sqrt{2}}{16}\exp\left(2\pi i\frac{z_{k}t}{16}\right)|t\rangle . \end{array}$$

Let *A* = *MN*/*r* − 1; transform formula (13) to

(15) $$\begin{array}{c} |\varphi \rangle =\frac{1}{\sqrt{\rho MN}}\overset{\rho MN}{\underset{k=1}{\sum }}|z_{k}\rangle =\frac{\sqrt{r}}{\sqrt{MN}}\overset{A}{\underset{j=0}{\sum }}|jr+l\rangle , \end{array}$$

where  *l* is the additional phase. Finally, formula (13) is measured and the probability state is selected which is |*t*〉 = |*k* *MN*/*r*〉 (*k* = 0,1,…, *r* − 1, *t* = *k* · *MN*/*r*). |*t*〉 represents the positions of all white points, so we extract all the positions of white points of palmprint, and then we can reconstruct the normalization grayscale of palmprint. Then we can extract the characteristic parameters *t* and finish the feature extraction step.

## 4. Palmprint Matching Algorithm Based on Grover Algorithm and Quantum Set Operations

### 4.1. Palmprint Matching Processing

Palmprint feature matching algorithm matches between identifying palmprint characteristics and registered palmprint characteristics in signature database; this algorithm makes the final identification decision on the basis of feature extraction. Finally we can determine the identity of a person. The most important part of this process is to select the appropriate feature matching strategy [20]. Assume that the identifying palmprint feature vectors are *A*_*i*_, a palmprint feature vector in the database is *B*_*i*_, *i* = (0,…, *l*), and *l* is the dimension of feature vectors. Then the Hamming distance between the two feature vectors is [21]

(16) $$\begin{array}{c} HD=\frac{1}{l}\overset{l}{\underset{i=1}{\sum }}A_{i}⊕B_{i}, \end{array}$$

where  ⊕ represents the exclusive or operation between two vectors. When the two vectors *A* and *B* are not the same, the result is 1; otherwise the result is 0. When we calculate all the elements in the vector, the dimension size of these two palmprint feature vectors is very large and calculation efficiency is very low. Therefore, in this paper, we propose a matching method by using Grover algorithm and quantum set operations. This method can ensure the matching accuracy and at the same time greatly improves the matching efficiency.

We assume that the identifying palmprint after the characteristics extraction has many feature vectors which are *A* = {*a*_0_, *a*_1_,…, *a*_*N*−1_} and *N* = 2^*n*^. If *N* ≠ 2^*n*^, we add the feature vector number of the identifying palmprint and let the result be *N* = 2^*n*^. By the same way, we assume that the feature vectors of palmprint image in database are *M* = 2^*m*^. The matching function in our method is defined as [22]

(17) $$\begin{array}{c} f_{c}\left(a_{i},b_{j}\right)=\{\begin{array}{cc} 1, & a_{i}=b_{j},\\ 0, & \text{else}. \end{array} \end{array}$$

To see whether the two palmprints are match or not is equivalent to calculate the intersection. The two records *a*_*i*0_ and *b*_*j*0_ are found out in a feature vector set which meets *a*_*i*0_ = *b*_*j*0_, that is, to calculate formula (17) which is *f*_*c*_(*a*_*i*_, *b*_*j*_) = 1.

We treat the identifying palmprint feature vector *A* as a database and store it in a memory. Each of the vectors *a*_*i*_ in the database corresponds to only one index *i*. The same method is used to deal with the palmprint vector *B*. Each of the vectors *b*_*j*_ corresponds only to one index *j*. Then we structure five registers which are shown in formula (18); five registers, respectively, save the index *i*, index *j*, vector *a*_*i*_, vector *b*_*j*_, and matching function value *f*_*c*_. Five registers are shown by the following equation:

(18) $$\begin{array}{c} {|i\rangle }_{\text{register}1}{|j\rangle }_{\text{register}2}{|a_{j}\rangle }_{\text{register}3}{|b_{j}\rangle }_{\text{register}4}{|f_{c}(a_{i},b_{j})\rangle }_{\text{register}5}. \end{array}$$

All the five registers are initialized as

(19) $$\begin{array}{c} {|0\rangle }_{\text{register}1}{|0\rangle }_{\text{register}2}{|0\rangle }_{\text{register}3}{|0\rangle }_{\text{register}4}{|0\rangle }_{\text{register}5}. \end{array}$$

Hadamard transform is applied to register1 and register2, and the effect is shown as

(20) H:|0〉|0〉|0〉|0〉|0〉 ⟶1MN(∑i=0N−1 ∑j=0M−1|i〉|j〉|0〉|0〉|0〉).

Then *U*_*L*_ is used as a unitary operation; the feature vectors of identifying palmprint and the palmprint from database are loaded in the quantum entanglement state, that is, to transform formula (20) into the following quantum state:

(21) $$\begin{array}{c} \frac{1}{\sqrt{MN}}\left(\overset{N-1}{\underset{i=0}{\sum }}\overset{M-1}{\underset{j=0}{\sum }}|i\rangle |j\rangle |a_{j}\rangle |b_{j}\rangle |0\rangle \right). \end{array}$$

A unitary operation is used in formula (22) after computing the matching function *f*_*c*_ between the two palmprints which are shown by the following equation:

(22) $$\begin{array}{c} \frac{1}{\sqrt{MN}}\left(\overset{N-1}{\underset{i=0}{\sum }}\overset{M-1}{\underset{j=0}{\sum }}|i\rangle |j\rangle |a_{j}\rangle |b_{j}\rangle |f_{c}\left(a_{i},b_{j}\right)\rangle \right). \end{array}$$

**Figure 7:**
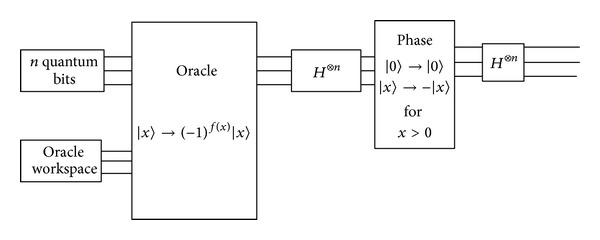
Quantum circuit of Grover algorithm.

The next step is to apply Grover algorithm in searching out the location of the matching palmprint in database. The quantum circuit of Grover algorithm [23] is shown in Figure 7.

The simulation platform is based on MATLAB; we add QCL (quantum computation language) as a toolbox in MATLAB to simulate quantum algorithms. There are lots of basic operations in quantum algorithms in QCL toolbox. QCL is a high level, architecture-independent programming language for quantum computers, with a syntax derived from classical procedural languages like C. This allows for the complete implementation and simulation of quantum algorithms. The key iterative operation of Grover is shown in Figure 7 (see Pseudocode 1).

**Pseudocode 1:**
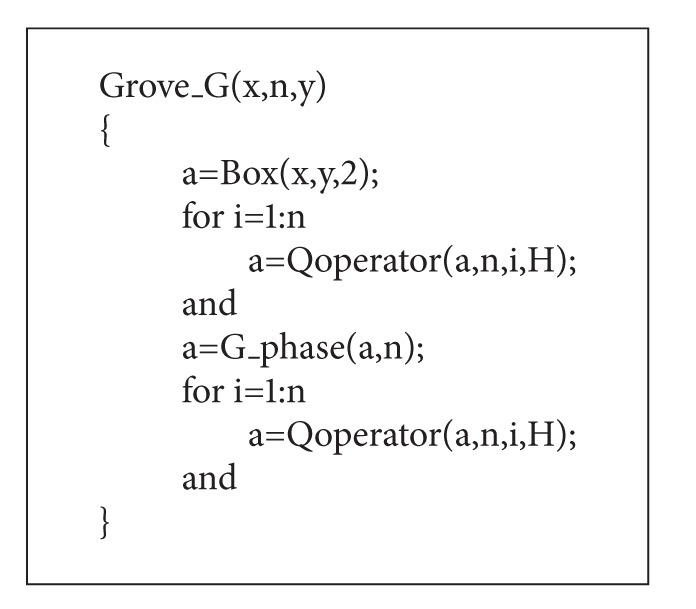


**Figure 8:**
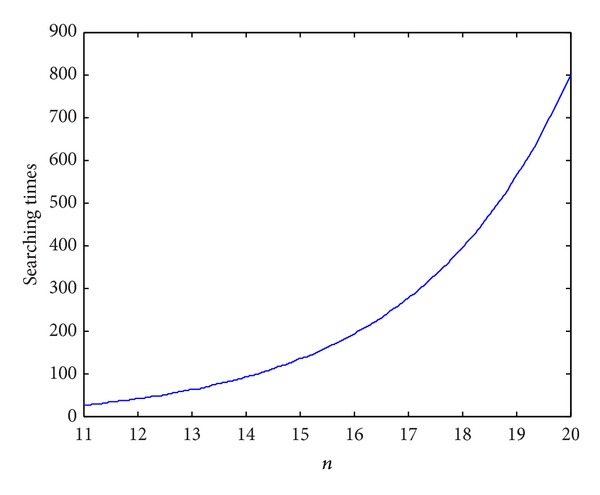
Relationship between Grover searching times and the number of quantum bits.

The number of identifying palmprints is 1, *n* quantum bits, size of searching space is *N* = 2^*n*^, and the relationship between Grover searching times and the number of quantum bits is shown in Figure 8.

Rossi et al. constructed a link between these initial states and hypergraphs, which provides an illustration of their entanglement properties [24]. In [25], the flow of quantum search algorithm and the quantum procedure model are shown. And the iteration steps in different quantum search problems are analyzed. Oracle operator is used to flip the phase of target quantum state. The effect is

(23) 1MN(∑i=0N−1∑j=0M−1(−1)fc(ai,bj)|i〉|j〉×|aj〉|bj〉|fc(ai,bj)〉register5).

From formula (17) we know that, when the two characteristic vectors are the same, formula (17) is equal to 1. Therefore, when the target palmprint is searched out, the corresponding quantum state will be reversed. And then the unitary transformation matrix *D* is used on the probability amplitude vector of all states; average of all the states' values is rotated, and matrix *D* can enlarge the probability of quantum state amplitude and greatly reduce the probability of finding out the target quantum states. Matrix *D* is defined as

(24) $$\begin{array}{c} Dpq=\{\begin{array}{cc} \frac{2}{N}, & p\neq q,\\ -1+\frac{2}{N}, & p=q. \end{array} \end{array}$$

Matrix form is expressed as

(25) $$\begin{array}{c} |\begin{array}{ccccc} -1+\frac{2}{N} & \frac{2}{N} & \ldots & \frac{2}{N} & \frac{2}{N}\\ \frac{2}{N} & -1+\frac{2}{N} & \ldots & \frac{2}{N} & \frac{2}{N}\\ \vdots & \vdots & \vdots & \vdots & \vdots \\ \frac{2}{N} & \frac{2}{N} & \ldots & -1+\frac{2}{N} & \frac{2}{N}\\ \frac{2}{N} & \frac{2}{N} & \ldots & \frac{2}{N} & -1+\frac{2}{N} \end{array}|. \end{array}$$

Compared to 2^*n*^ times of calculation in traditional method, we just need to apply *O*(*N* ^1/2^) times of operation from formula (23) to formula (25). Then we measure register1 and the location of the target iris can be found out from palmprint database.

### 4.2. Palmprint Matching Algorithm Experiment and Analysis

Simulation experiment result is based on the standard library Poly U; Poly U palmprint image library is one of the largest image libraries in palmprint recognition public field. Poly U library has 600 images from 100 people (everyone has 6 images). The experiment is divided into the same palmprint experiment and cross-validation experiment.

In the same palmprint experiment, we extract 64 palmprints and put them in a database; each of the 64 palmprints is from different people. A palmprint in database is selected as an identifying palmprint. We use speed and accuracy to compare our Grover algorithm and quantum set operations with traditional Euclidean distance method. The same palmprint experiment results are shown in Table 1.

In the cross-validation experiment, we extract 64 palmprints which are different from above palmprints and put them in a database; each of the 64 palmprints is from different people. In the cross-validation experiment, we first estimate the number of matched palmprints. We can estimate the number of solutions much more quickly than by traditional method by combining the Grover interation with the phase estimation method.

**Figure 9:**
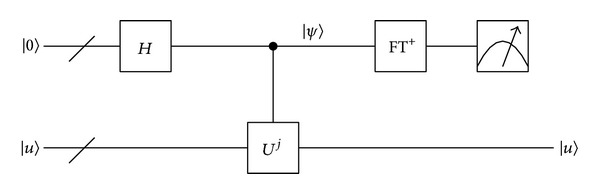
Schematic of the overall phase estimation procedure.

The essence of Grover searching algorithm is to determinate the phase flip angle; if the angle is known, we can get the number of matched palmprints. In this paper we use phase estimation method to identify the phase of target quantum state. Phase estimation method is needed to build two registers. An overall schematic of the algorithm is shown in Figure 9.

The top line (the “/” denotes a bundle of wires) is the first register; the bottom line is the second register. Unitary operator *U* has an eigenvector |*u*〉 with eigenvalue *e* ^2*πiφ*^; this schematic is used to estimate *φ*. FT^+^ is inverse QFT.

First we prepare the state |*u*〉; then we apply a Hadamard transform to the first register, followed by application of controlled *U* operations on the second register, with *U* raised to successive powers of two, *U* ^*j*^ = *U* ^2^*i*^^(*i* = 0,1,…, *t* − 1). The final state of the first register is easily seen to be

(26) |ψ〉=12t/2(|0〉+e2πi2t−1φ|1〉) ×(|0〉+e2πi2t−2φ|1〉)⋯(|0〉+e2πi20φ|1〉)=12t/2∑k=02t−1e2πiφk|k〉;

then we apply the inverse QFT on the quantum state |*ψ*〉:

(27) $$\begin{array}{c} \frac{1}{2^{t/2}}\overset{2^{t}-1}{\underset{j=0}{\sum }}e^{2\pi i\varphi j}|j\rangle |u\rangle ⟶|\tilde{\varphi }\rangle |u\rangle . \end{array}$$

After we measure the first register, we can get the estimate value $|\tilde{\varphi }\rangle$ of *φ*.

We use phase estimation method to estimate the phase when we search in training palmprints. Then we use Grover to estimate the number of palmprint which is matched with the validation palmprint. The cross-validation experiment results are shown in Table 2.

In Table 1, we can get that the matching numbers of our algorithm which combines the quantum set operations with Grover algorithm only need 8 times, that the traditional algorithm which calculates the Euclidean distance will need 64 times, and that our method has square root of the traditional one. The quantum set operations and Grover matching method needs 0.20 s matching time which has an obvious advantage on efficiency. Additionally, our matching accuracy is almost 100% which is better than Euclidean distance calculation method. In Table 2, by using the cross-validation experiment, we can see that quantum set operations and Grover matching method has good performance with short matching time, which can save more space and time in processing and will be more suitable in applications. We can get higher matching accuracy by using quantum algorithm compared with traditional algorithm too.

## 5. Conclusions

Quantum algorithms are applied to palmprint recognition in this paper. Palmprint is filtered by using quantum adaptive median filtering algorithm. Compared with the traditional methods, we can see from the filtering effect chart that this method possesses an enhanced ability of filtering and in the meantime conserves palmprint details. Then, the features of palm print are extracted by means of QFT; the features of pattern can be obtained via quantum parallel characteristics. Analysis shows that the pace of our quantum algorithms has increased exponentially compared with the pace of traditional feature extraction algorithm. Eventually, palmprint matching processing is carried out by using Grover algorithm and quantum set operations. As you can see from the analysis of the experimental result, quantum algorithms can increase matching accuracy and shorten the matching time. Therefore, it gets a marvelous matching effect.

## Figures and Tables

**Table 1:** The same palmprint experiment results of traditional algorithm and our algorithm.

Algorithm	Euclidean distance calculation method	Quantum set operations and Grover algorithm
Matching numbers	64	8
Matching time (s)	0.41	0.20
Matching accuracy (%)	94	99

**Table 2:** Cross-validation experiment results of traditional algorithm and our algorithm.

Algorithm	Euclidean distance calculation method	Quantum set operations and Grover algorithm
Matching numbers	64	8
Matching time (s)	0.52	0.31
Matching accuracy (%)	93	99
